# Compression-induced improvements in post-exercise recovery are associated with enhanced blood flow, and are not due to the placebo effect

**DOI:** 10.1038/s41598-022-21029-2

**Published:** 2022-10-06

**Authors:** Shane F. O’Riordan, David J. Bishop, Shona L. Halson, James R. Broatch

**Affiliations:** 1 Institute for Health and Sport (iHeS), Victoria University, PO Box 14428, Melbourne, VIC 8001 Australia; 2 Department of Physiology, Australian Institute of Sport, Canberra, Australia; 3 School of Behavioural and Health Sciences, Australian Catholic University, Brisbane, Australia

**Keywords:** Physiology, Health occupations

## Abstract

The aim of this study was to investigate the physiological effects of compression tights on blood flow following exercise and to assess if the placebo effect is responsible for any acute performance or psychological benefits. Twenty-two resistance-trained participants completed a lower-body resistance exercise session followed by a 4 h recovery period. Participants were assigned a post-exercise recovery intervention of either compression tights applied for 4 h (COMP), placebo tablet consumed every hour for 4 h (PLA) or control (CON). Physiological (markers of venous return, muscle blood flow, blood metabolites, thigh girth), performance (countermovement jump, isometric mid-thigh pull), and psychological measures (perceived muscle soreness, total quality of recovery) were collected pre-exercise, immediately post-exercise, at 30 (markers of venous return and muscle blood flow) and 60 min (blood metabolites, thigh girth and psychological measures) intervals during 4 h of recovery, and at 4 h, 24 h and 48 h post-exercise. No significant (*P* > 0.05) differences were observed between interventions. However, effect size analysis revealed COMP enhanced markers of venous return, muscle blood flow, recovery of performance measures, psychological measures and reduced thigh girth compared to PLA and CON. There were no group differences in blood metabolites. These findings suggest compression tights worn after resistance exercise enhance blood flow and indices of exercise recovery, and that these benefits were not due to a placebo effect.

## Introduction

Exercise-induced muscle damage (EIMD) frequently occurs following unaccustomed exercise^1^, particularly if it is comprised of a large eccentric component. The etiology of EIMD is characterised by structural damage to the myofibrils during the initial exercise insult, followed by secondary inflammation from leukocyte infiltration into the damaged tissue^2,3^. The signs and symptoms associated with EIMD include muscle soreness, muscle swelling, reduced muscle function, and elevated concentration of myofibrillar proteins in the blood (e.g., creatine kinase (CK), lactate dehydrogenase (LDH))^2–5^. These can become evident within a few hours after exercise^3^ and can persist for several days^2^. Although EIMD is an important process for the adaptive response to exercise training^4^, reducing the symptoms associated with EIMD is beneficial for individuals aiming to maintain short-term exercise performance and training quality^6^. Post-exercise recovery strategies are commonly utilised to alleviate the symptoms of EIMD^7^, with one of the most prevalent techniques being sports compression garments (SCG)^8^.

The mechanisms by which SCG enhance recovery following exercise remain unclear but may be closely associated with venous and muscle blood flow alterations^9^. Compression-induced increases in venous blood flow^9^ is linked with accelerating the removal of myofibrillar proteins from the muscle^10,11^. For example, lower concentrations of plasma CK have been reported with compression garment use during the post-exercise recovery^12,13^. Compression may also reduce exercise-induced oedema by limiting the space available for swelling to form and by promoting lymphatic outflow, thus attenuating the inflammatory response and preventing further muscle damage^8,14^. In addition, compression may improve blood flow to the muscle following exercise and aid recovery by increasing nutrient delivery post-exercise. Nonetheless, a limitation with compression research to date is that most studies have focused on blood flow responses during exercise^15–19^, and within a short period (< 1 h) post-exercise^17,18^. Considering the inflammatory response begins in the early hours (1–4 h) during exercise recovery^5^, the use of compression garments beyond the first hour post-exercise may serve to reduce the symptoms of EIMD. The assessment of compression-induced changes in blood flow during this time will provide valuable insight into the proposed mechanism (i.e., increased blood flow) attributed to the effectiveness of SCG as a post-exercise recovery strategy.

A further limitation of the compression research to date is that the contribution of psychological factors to the ergogenic effects of compression on exercise performance and recovery outcomes is currently unknown. Considering many studies report favourable psychological outcomes (e.g., perceived muscle soreness, quality of recovery) with a concomitant lack of effect on physiological and/or performance measures^20–22^, it is possible that psychological factors may contribute, at least in part, to the benefits associated with compression^23^. In further support of this, participants’ positive perception and belief in SCG are suggested to improve exercise performance and recovery^21,24,25^. Research in other recovery techniques, including massage^26^ and cold water immersion^27^, suggest enhanced exercise recovery might occur via psychophysiological mechanisms (i.e., placebo effect)^27^; however, this has not been appropriately investigated with sports compression garments.

Due to the high level of pressure exerted on the limb with compression garments, a challenge with compression research is the inability to blind participants. Previous research has attempted to blind participants using similar-looking garments with a low level of pressure^28,29^. Alternatively, compression research has used 'sham' placebo interventions (e.g., drink^30^ and ultrasound^20,31,32^), in which participants were deceived into thinking these interventions are beneficial for exercise performance and/or recovery. However, a limitation of these studies is that belief in the placebo intervention (as compared with compression) was not assessed, meaning the presence of a placebo effect is still unable to be discounted^20,30–32^. In addition, no study has incorporated a placebo intervention promoting the beneficial effect on blood flow, despite the general consensus that the ergogenic effects associated with SCG are closely related to improvements in blood flow^8,11,33^. In order to determine the effectiveness of SCG on exercise recovery, it is crucial to assess this placebo effect.

This study aimed to investigate the influence of sports compression tights on post-exercise recovery and markers of blood flow compared to those of a placebo condition that participants were informed was as effective as compression for the recovery from exercise. We hypothesised that sports compression tights would enhance post-exercise blood flow and subsequently improve indices of muscle recovery, and that these benefits would not be related to the placebo effect—i.e., compression would elicit superior benefits when compared with the placebo and control conditions.

## Results

A detailed summary of statistical data for all between-group effects for blood flow measures (markers of venous return and muscle blood flow), performance measures (CMJ and IMTP), and perceptual/swelling measures (MS, TQR and thigh girth) are presented in Tables 1, 4 and 6, respectively.

**Table 1:** Summary of all between-group effects for muscle blood flow and markers of venous return.

Measure	Group comparison	Change between	Mean difference in change		Standardised ES		Effect magnitude
			Absolute difference	± 95% CI	ES	± 95% CI	
Popliteal CSA (cm^2^)	COMP vs. CON	PRE—240 min	0.05	0.04	0.49	0.39	Small
Popliteal V_mean_ (cm/s)	COMP vs. CON	PRE—90 min	0.85	1.66	0.58	1.17	Medium
		PRE—180 min	1.02	1.61	0.71	1.08	Medium
		PRE—210 min	0.76	1.72	0.52	1.28	Medium
		PRE—240 min	0.73	2.17	0.62	1.65	Medium
	COMP vs. PLA	PRE—30 min	1.17	1.25	2.21	2.88	Large
		PRE—90 min	0.53	0.69	1.05	1.29	Large
		PRE—120 min	0.48	1.23	1.05	2.34	Large
		PRE—180 min	0.59	1.11	1.35	2.39	Large
		PRE—210 min	0.78	1.02	1.69	2.07	Large
		PRE—240 min	0.89	0.93	1.95	1.91	Large
Popliteal V_peak_ (cm/s)	COMP vs. PLA	PRE—30 min	3.26	4.58	0.98	1.64	Large
		PRE—240 min	1.60	4.35	0.73	1.65	Medium
Popliteal blood flow (mL/min)	COMP vs. PLA	PRE—30 min	45.31	53.85	0.64	0.79	Medium
Femoral CSA (cm^2^)	COMP vs. CON	PRE—120 min	0.14	0.29	0.54	1.05	Medium
		PRE—150 min	0.14	0.25	0.56	0.92	Medium
Femoral V_mean_ (cm/s)	COMP vs. CON	PRE—60 min	2.56	3.52	1.50	1.77	Large
		PRE—90 min	2.52	3.75	1.28	1.79	Large
		PRE—120 min	1.89	2.07	0.94	1.77	Large
		PRE—150 min	2.93	2.83	1.55	1.51	Large
		PRE—180 min	1.80	2.02	1.09	1.20	Large
		PRE—210 min	2.04	1.99	1.15	1.05	Large
		PRE—240 min	1.42	1.86	0.72	1.11	Medium
	COMP vs. PLA	PRE—60 min	1.64	3.48	1.02	1.93	Large
		PRE—120 min	1.09	2.37	0.60	1.33	Medium
		PRE—150 min	1.33	2.98	0.65	1.59	Medium
		PRE—180 min	1.32	1.69	0.80	2.14	Large
		PRE—210 min	1.46	3.02	0.91	1.90	Large
Femoral V_peak_ (cm/s)	COMP vs. CON	PRE—60 min	4.26	12.19	0.60	1.31	Medium
		PRE—150 min	8.27	8.64	0.82	1.06	Large
		PRE—180 min	4.01	6.56	0.49	0.92	Medium
		PRE—210 min	3.89	10.7	0.47	1.05	Medium
		PRE—240 min	5.07	8.44	0.52	1.03	Medium
Femoral blood flow (mL/min)	COMP vs. CON	PRE—60 min	229.55	444.11	1.01	1.65	Large
		PRE—90 min	258.03	399.59	0.94	1.65	Large
		PRE—120 min	200.49	178.56	0.87	0.93	Large
		PRE—150 min	223.14	249.86	1.24	1.24	Large
		PRE—180 min	211.04	198.19	1.07	0.96	Large
		PRE—210 min	215.80	217.91	1.03	0.95	Large
		PRE—240 min	150.10	158.46	0.61	0.79	Medium
	COMP vs. PLA	PRE—180 min	97.16	170.47	0.76	0.95	Medium
		PRE—210 min	110.86	220.43	0.61	1.22	Medium
Muscle blood flow (mL/min·100/g)	COMP vs. CON	PRE—30 min	0.07	0.08	0.79	0.96	Medium
		PRE—60 min	0.07	0.07	0.93	0.75	Large
		PRE—90 min	0.07	0.06	1.01	0.94	Large
		PRE—150 min	0.05	0.05	0.78	0.85	Medium
		PRE—180 min	0.05	0.04	0.88	0.86	Large
		PRE—210 min	0.06	0.05	1.15	0.80	Large
		PRE—240 min	0.07	0.04	1.03	0.53	Large
	COMP vs. PLA	PRE—30 min	0.07	0.08	0.55	0.97	Medium
		PRE—60 min	0.06	0.07	0.54	0.81	Medium
		PRE—90 min	0.06	0.06	0.78	0.87	Medium
		PRE—150 min	0.04	0.06	0.44	0.68	Small
		PRE—180 min	0.04	0.05	0.65	0.71	Medium
		PRE—210 min	0.04	0.05	0.60	0.70	Medium

### Garment details

The compression tights applied 14.0 ± 2.2 mmHg, 17.4 ± 2.6 mmHg, 20.1 ± 2.5 mmHg, 13.9 ± 2.5 mmHg, 13.9 ± 2.5 mmHg and 12.6 ± 2.4 mmHg of pressure to the lower-limb of COMP group at landmarks A (upper ankle) to F (upper thigh), respectively.

### Belief questionnaires

The average belief in the interventions for all participants was 3.3 ± 0.7 for COMP, and 3.3 ± 0.9 for PLA. Participants assigned to COMP had a baseline belief in their intervention of 3.0 ± 0.6, which was significantly lower (*P* = 0.037) than participants in the PLA condition (3.9 ± 0.8). Belief in the COMP intervention (3.9 ± 0.8) was significantly higher (*P* = 0.040) than that of the PLA intervention (3.0 ± 0.5) at the end of the study.

### Popliteal markers of venous return

**Figure 1:**
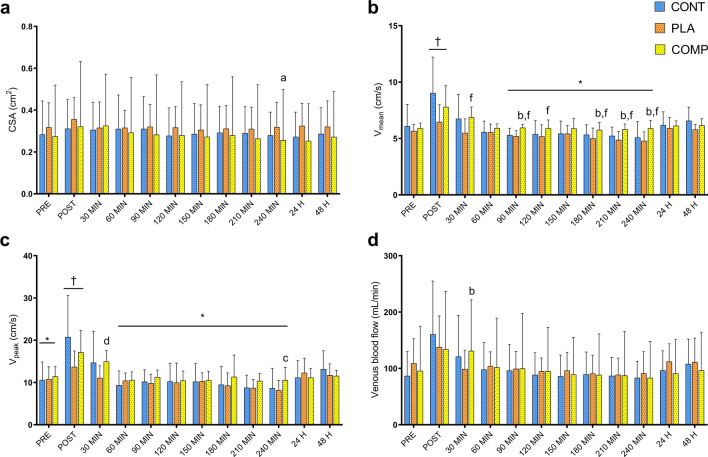
Popliteal vein markers of venous return for CON, PLA and COMP conditions. Markers measured include (**a**) cross-sectional area (CSA), (**b**) mean flow velocity (V_mean_), (**c**) peak flow velocity (V_peak_) and (**d**) venous blood flow. Time points are before exercise (PRE), immediately post-exercise (POST), 4 h recovery (30–240 min), and 24 and 48 h post-exercise. All data presented as mean ± SD. ^†^Significant time effect as compared with all other time points. *Significant time effect as compared with 30 min post-exercise. ^a^Small effect as compared with CON. ^b^Medium effect as compared with CON. ^e^Medium effect as compared with PLA. ^f^Large effect as compared with PLA.

There were no interaction effects for popliteal CSA (*F*_22, 228_ = 0.30, *P* = 1.000; Fig. 1a), V_mean_ (*F*_22, 228_ = 0.94, *P* = 0.543; Fig. 1b), V_peak_ (*F*_22, 228_ = 0.78, *P* = 0.766; Fig. 1c), and venous blood flow (*F*_22, 228_ = 0.14, *P* = 1.000; Fig. 1d), or main effects of time for popliteal CSA (*F*_*11, 228*_ = 0.13, *P* = 1.000) and venous blood flow (*F*_*11, 228*_ = 1.54, *P* = 0.110). There were main effects of time for elevated popliteal V_mean_ (*F*_*11, 228*_ = 8.01, *P* < 0.001; Fig. 1b) and V_peak_ (*F*_*11, 228*_ = 7.08, *P* < 0.001; Fig. 1c) post-exercise. Effect size analysis revealed medium to large effects for increased popliteal V_mean_ during the 4-h post-exercise recovery in the COMP group compared to the CON and PLA groups, respectively (Table 1).

### Femoral markers of venous return

**Figure 2:**
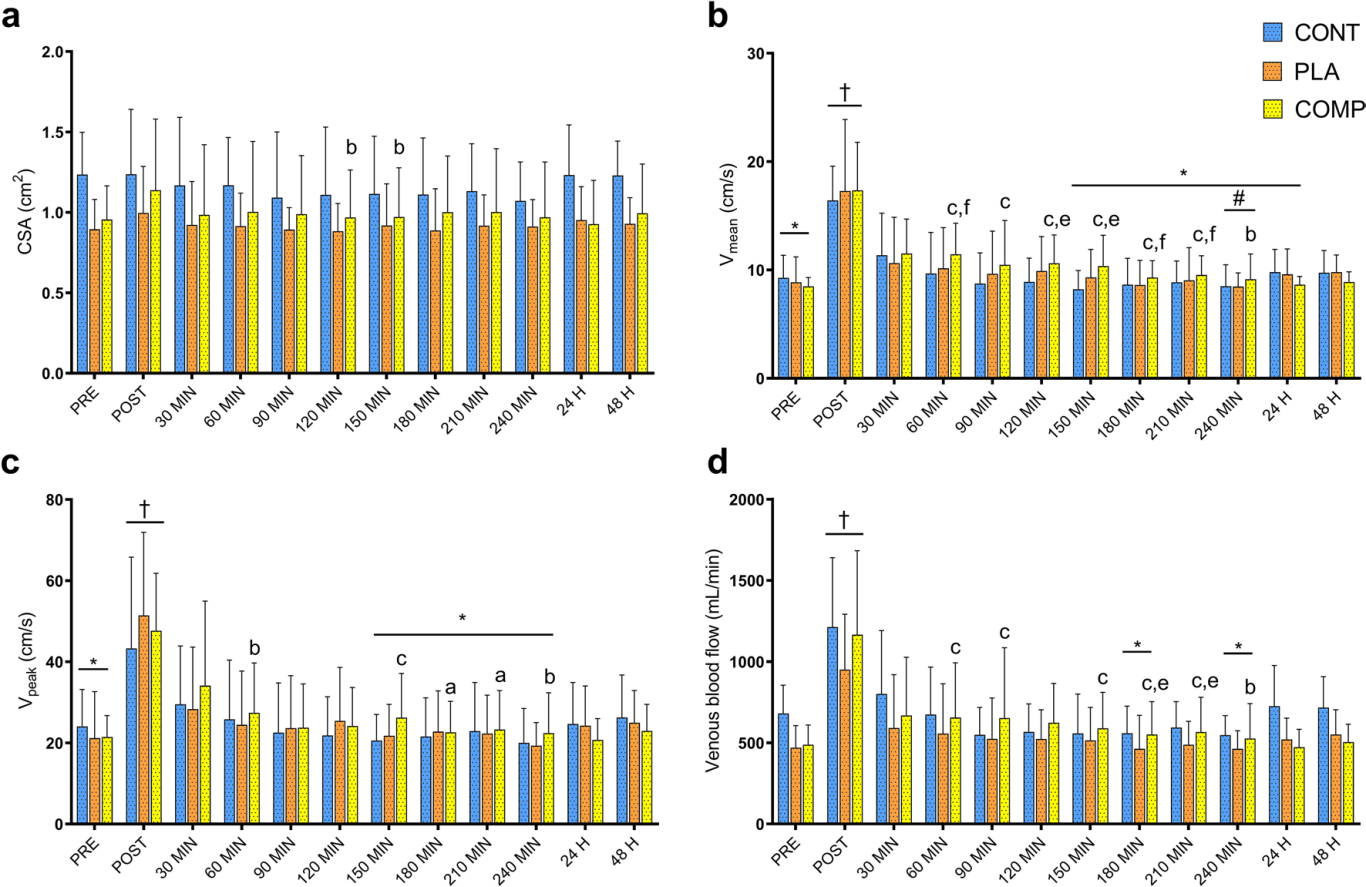
Common femoral vein markers of venous return for CON, PLA and COMP conditions. Markers measured include (**a**) cross-sectional area (CSA), (**b**) mean flow velocity (V_mean_), (**c**) peak flow velocity (V_peak_) and (**d**) venous blood flow. Time points are before exercise (PRE), immediately post-exercise (POST), 4 h recovery (30–240 min), and 24 and 48 h post-exercise. All data presented as mean ± SD. ^†^Significant time effect as compared with all other time points. *Significant time effect as compared with 30 min post-exercise. ^#^Significant time effect as compared with 60 min post-exercise. ^a^Small effect as compared with CON. ^b^Medium effect as compared with CON. ^c^Large effect as compared with CON. ^e^Medium effect as compared with PLA. ^f^Large effect as compared with PLA.

There were no interaction effects for femoral CSA (*F*_22, 228_ = 0.09, *P* = 1.000; Fig. 2a), V_mean_ (*F*_22, 228_ = 0.27, *P* = 1.000; Fig. 2b), V_peak_ (*F*_22, 228_ = 0.13, *P* = 1.000; Fig. 2c), and venous blood flow (*F*_22, 228_ = 0.39, *P* = 0.966; Fig. 2d), or main effects of time for femoral CSA (*F*_11, 228_ = 0.33, *P* = 0.984). There were main effects of time for elevated femoral V_mean_ (*F*_11, 228_ = 11.39, *P* < 0.001; Fig. 2b), V_peak_ (*F*_11, 228_ = 7.05, *P* < 0.001; Fig. 2c,) and venous blood flow (*F*_11, 228_ = 6.91, *P* < 0.001; Fig. 2d) post-exercise. Effect size analysis revealed medium to large effects for increased V_mean_ and venous blood flow during the 4-h post-exercise recovery in the COMP group compared to the CON and PLA groups (Table 1).

### Muscle blood flow

**Figure 3:**
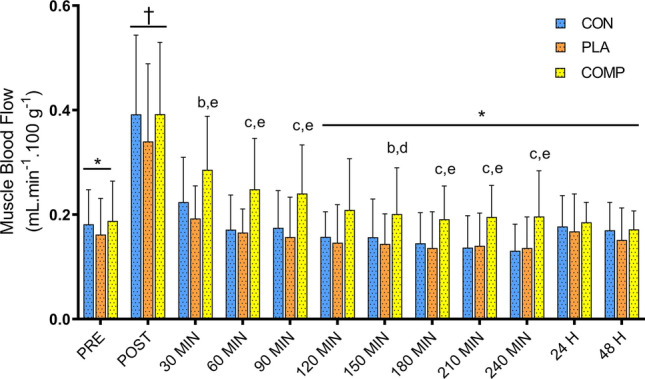
Muscle blood flow (millilitres of blood per min per 100 g of tissue) for CON, PLA and COMP conditions. Time points are before exercise (PRE), immediately post-exercise (POST), 4 h recovery (30–240 min), and 24 and 48 h post-exercise. All data presented as mean ± SD. ^†^Significant time effect as compared with all other time points. *Significant time effect as compared with 30 min post-exercise. ^b^Medium effect as compared with CON. ^c^Large effect as compared with CON. ^d^Small effect as compared with PLA. ^e^Medium effect as compared with PLA.

There was no interaction effect for muscle blood flow (*F*_22, 228_ = 0.28, *P* = 1.000). There were main effects of time (*F*_11, 228_ = 11.94, *P* < 0.001) for muscle blood flow (Fig. 3). Specifically, muscle blood flow was elevated post-exercise compared to all other time points (*P* < 0.001) and elevated at 30 min as compared with baseline and 120 min, 150 min, 180 min, 210 min, 240 min, 24 h, and 48 h post-exercise (*P* < 0.001). Effect size analysis revealed small to large effects for increases in muscle blood flow during the 4-h post-exercise recovery in the COMP group compared to the CON and PLA groups (Table 1).

### Performance

There were no interaction (*F*_*8, 95*_ < 1.00, *P* > 0.05) or main effects of time (*F*_*4, 95*_ < 1.00, *P* > 0.05) for all CMJ (Table 2) and IMTP (Table 3) variables measured. For CMJ variables, there were small to large effects for improved performance post-exercise in the COMP group compared to the CON and PLA groups (Table 4). There were also medium effects for improved IMTP performance at 4 h post-exercise in the COMP group compared to the CON and PLA groups (Table 4).

**Table 2:** Countermovement jump (CMJ) measures for CON, PLA and COMP conditions.

Variable	Condition	Time point				
		PRE	POST	4 h	24 h	48 h
Jump height (m)	CON	0.28 ± 0.04	0.26 ± 0.03	0.25 ± 0.02	0.27 ± 0.03	0.27 ± 0.03
	PLA	0.28 ± 0.07	0.25 ± 0.06	0.25 ± 0.06	0.26 ± 0.07	0.27 ± 0.07
	COMP	0.31 ± 0.06	0.29 ± 0.06	0.30 ± 0.06^a,d^	0.31 ± 0.06 ^a^	0.31 ± 0.06^a^
Relative peak force (N/kg)	CON	22.8 ± 1.6	21.8 ± 1.9	21.4 ± 1.3	21.7 ± 1.7	21.7 ± 1.6
	PLA	23.0 ± 3.1	22.4 ± 3.8	22.2 ± 2.5	22.1 ± 2.2	22.5 ± 2.3
	COMP	21.6 ± 1.0	21.2 ± 1.3	21.0 ± 0.9^b^	21.8 ± 0.8^c,d^	21.5 ± 1.0 ^b^
Relative peak power (W/kg)	CON	43.7 ± 3.8	41.4 ± 3.1	40.5 ± 2.9	41.9 ± 2.5	42.8 ± 2.9
	PLA	47.4 ± 9.0	44.6 ± 7.9	44.3 ± 7.4	45.4 ± 7.6	46.6 ± 7.1
	COMP	48.3 ± 5.5	46.1 ± 6.5	46.6 ± 5.3^a^	48.5 ± 5.7^a,d^	48.4 ± 5.7
Total duration (s)	CON	0.64 ± 0.04	0.68 ± 0.09	0.66 ± 0.08	0.65 ± 0.09	0.65 ± 0.08
	PLA	0.63 ± 0.07	0.65 ± 0.10	0.66 ± 0.08	0.65 ± 0.08	0.65 ± 0.09
	COMP	0.64 ± 0.07	0.67 ± 0.09	0.66 ± 0.08^b,e^	0.64 ± 0.07^c,f^	0.64 ± 0.08^e^

Measures include jump height, relative peak force, relative peak power and total duration. Time points are before exercise (PRE), immediately post-exercise (POST), and 4, 24, and 48 h post-exercise. All data presented as mean ± SD.

^a^Small effect as compared with CON.

^b^Medium effect as compared with CON.

^c^Large effect as compared with CON.

^d^Small effect as compared with PLA.

^e^Medium effect as compared with PLA.

^f^Large effect as compared with PLA.

**Table 3:** Isometric mid-thigh pull (IMTP) measures for CON, PLA and COMP conditions.

Variable	Condition	Time point				
		PRE	POST	4 h	24 h	48 h
Peak force (N)	CON	1467 ± 447	1309 ± 314	1329 ± 337	1305 ± 364	1304 ± 382
	PLA	1241 ± 374	1228 ± 444	1202 ± 397	1149 ± 382	1148 ± 442
	COMP	1621 ± 290	1615 ± 380	1535 ± 292	1513 ± 267	1462 ± 444
Relative peak force (N/kg)	CON	19.6 ± 3.3	17.9 ± 3.9	18.0 ± 3.2	17.7 ± 3.8	17.5 ± 3.6
	PLA	16.3 ± 3.9	15.8 ± 4.1	15.7 ± 4.0	14.9 ± 3.9	14.9 ± 5.1
	COMP	21.5 ± 1.4	21.2 ± 2.1	20.4 ± 2.2	20.2 ± 1.9	19.1 ± 3.6
Force at 100 ms (N)	CON	353 ± 204	323 ± 136	348 ± 78	352 ± 125	284 ± 73
	PLA	285 ± 148	287 ± 98	342 ± 233	317 ± 184	292 ± 149
	COMP	358 ± 196	381 ± 109	495 ± 102^a,b^	436 ± 127	378 ± 148
Force at 200 ms (N)	CON	658 ± 251	618 ± 185	665 ± 179	640 ± 176	559 ± 135
	PLA	595 ± 248	588 ± 233	595 ± 347	579 ± 260	547 ± 232
	COMP	752 ± 244	771 ± 175	930 ± 215^a,b^	804 ± 180	705 ± 232
RFD 0–100 ms (N/s)	CON	3096 ± 2043	2819 ± 1359	3064 ± 787	3090 ± 1250	2422 ± 739
	PLA	2455 ± 1480	2425 ± 989	2987 ± 2305	2735 ± 1814	2539 ± 1437
	COMP	3123 ± 1964	3309 ± 1141	4402 ± 1132^a,b^	3840 ± 1289	3295 ± 1436
RFD 0–200 ms (N/s)	CON	3075 ± 1254	2884 ± 926	3117 ± 896	2987 ± 876	2585 ± 677
	PLA	2777 ± 1245	2717 ± 1165	2759 ± 1722	2681 ± 1285	2545 ± 1129
	COMP	3534 ± 1222	3601 ± 887	4364 ± 1120^a,b^	3761 ± 915	3282 ± 1135

Measures include peak force, relative peak force, force at 100 ms and 200 ms, and rate of force development (RFD) at 100 ms and 200 ms. Time points are before exercise (PRE), immediately post-exercise (POST), and 4, 24, and 48 h post-exercise. All data presented as mean ± SD.

^a^Medium effect as compared with CON.

^b^Medium effect as compared with PLA.

**Table 4:** Summary of all between-group effects for CMJ and IMTP variables.

Measure	Group comparison	Change between	Mean difference in change		Standardised ES		Effect magnitude
			Absolute difference	± 95% CI	ES	± 95% CI	
CMJ jump height (m)	COMP vs. CON	PRE—4 h	0.02	0.02	0.45	0.32	Small
		PRE—24 h	0.01	0.02	0.29	0.27	Small
		PRE—48 h	0.02	0.01	0.29	0.19	Small
	COMP vs. PLA	PRE—4 h	0.02	0.02	0.27	0.21	Small
CMJ relative peak force (N/kg)	COMP vs. CON	PRE—4 h	0.80	1.07	0.56	0.78	Medium
		PRE—24 h	1.27	1.11	0.94	0.83	Large
		PRE—48 h	0.97	0.99	0.71	0.74	Medium
	COMP vs. PLA	PRE—24 h	1.11	1.47	0.48	0.63	Small
CMJ relative peak power (W/kg)	COMP vs. CON	PRE—4 h	1.62	2.15	0.37	0.41	Small
		PRE—24 h	1.99	1.97	0.39	0.39	Small
	COMP vs. PLA	PRE—24 h	2.40	2.17	0.31	0.26	Small
CMJ duration (s)	COMP vs. CON	PRE—4 h	0.04	0.06	0.76	1.17	Medium
		PRE—24 h	0.06	0.07	0.99	1.33	Large
	COMP vs. PLA	PRE—4 h	0.05	0.04	0.69	0.58	Medium
		PRE—24 h	0.06	0.04	0.81	0.55	Large
		PRE—48 h	0.05	0.05	0.66	0.63	Medium
IMTP force @ 100 ms (N)	COMP vs. CON	PRE—4 h	166.28	246.75	0.69	1.02	Medium
	COMP vs. PLA	PRE—4 h	80.55	199.99	0.60	0.97	Medium
IMTP force @ 200 ms (N)	COMP vs. CON	PRE—4 h	209.86	331.12	0.70	1.36	Medium
	COMP vs. PLA	PRE—4 h	168.43	307.87	0.66	1.08	Medium
IMTP RFD @ 100 ms (N/s)	COMP vs. CON	PRE—4 h	1152.04	2497.88	0.57	1.31	Medium
	COMP vs. PLA	PRE—4 h	746.72	2040.61	0.56	0.99	Medium
IMTP RFD @ 200 ms (N/s)	COMP vs. CON	PRE—4 h	983.69	1670.42	0.64	1.38	Medium
	COMP vs. PLA	PRE—4 h	801.69	1555.90	0.63	1.09	Medium

### Perceptual measures

There were no interaction effects for muscle soreness (*F*_14, 152_ = 0.75, *P* = 0.720) or TQR (*F*_14, 152_ = 0.53, *P* = 0.914). There were main effects of time for muscle soreness (*F*_7, 152_ = 15.70, *P* < 0.001) and TQR (*F*_*7, 152*_ = 14.37, *p* < 0.001). Specifically, muscle soreness was higher (*P* < 0.05), and TQR lower (*P* < 0.05), at every time point as compared with baseline (Table 5). For muscle soreness, there were medium to large effects for lower ratings post-exercise in the COMP group compared to CON and PLA groups (Table 6). There were also large effects for increased TQR at all time-points post-exercise in the COMP group compared to the CON and PLA groups (Table 6).

**Table 5:** Muscle soreness (MS), total quality of recovery (TQR), thigh girth, lactate dehydrogenase (LDH) and creatine kinase (CK) for CON, PLA and COMP conditions.

Variable	Condition	Time point							
		PRE^†^	POST	60 min $§	120 min**^$§^	180 min**^$§^	240 min**^$§^	24 h	48 h
MS (AU)	CON	0.9 ± 0.9	4.4 ± 1.4	3.4 ± 1.1	3.0 ± 0.6	2.7 ± 1.3	2.6 ± 1.0	5.3 ± 1.6	5.9 ± 1.6
	PLA	0.8 ± 1.0	3.9 ± 1.9	3.4 ± 1.8	3.0 ± 2.0	3.0 ± 2.0	3.4 ± 2.4	6.5 ± 1.5	5.5 ± 2.1
	COMP	0.6 ± 0.8	3.3 ± 2.4	3.3 ± 2.4	2.0 ± 1.9^a,b^	1.9 ± 1.6^c,e^	1.4 ± 1.6^c,f^	4.0 ± 1.5^f^	3.2 ± 1.9^c,f^

		PRE^†^	POST	60 min**^$^	120 min**^$^	180 min**^#$§^	240 min**^#$§^	24 h	48 h**
TQR (AU)	CON	17.9 ± 1.2	12.6 ± 1.1	13.6 ± 1.4	14.7 ± 1.4	15.3 ± 1.7	15.6 ± 2.1	12.0 ± 1.8	12.6 ± 2.5
	PLA	18.8 ± 1.5	13.0 ± 3.4	15.1 ± 2.7	15.6 ± 2.3	16.4 ± 2.2	16.6 ± 2.1	12.9 ± 2.2	14.8 ± 2.4
	COMP	17.9 ± 1.2	13.4 ± 1.3	16.0 ± 2.5 ^c,f^	16.7 ± 3.0 ^c,f^	17.3 ± 2.9 ^c,f^	17.7 ± 2.8 ^c,f^	14.7 ± 1.4 ^c,f^	16.2 ± 1.9 ^c,f^

		PRE	POST	60 min	120 min	180 min	240 min	24 h	48 h
Thigh girth (cm)	CON	53.9 ± 5.9	54.9 ± 6.1	54.6 ± 5.9	54.5 ± 5.9	54.4 ± 5.9	54.3 ± 5.9	55.3 ± 6.1	55.0 ± 6.1
	PLA	56.2 ± 4.2	57.3 ± 4.4	57.0 ± 4.3	56.8 ± 4.2	56.7 ± 4.1	56.7 ± 4.1	57.6 ± 4.4	57.4 ± 4.4
	COMP	54.5 ± 5.4	55.5 ± 5.5	53.6 ± 5.2^a,d^	53.6 ± 5.2^a,d^	53.6 ± 5.2^a,d^	53.6 ± 5.2 ^a,d^	55.1 ± 5.5	54.8 ± 5.4

		PRE	POST	60 min*^$§^	120 min*^$§^	180 min*^$§^	240 min*^$§^	24 h	48 h
LDH (U/L)	CON	81.2 ± 19.4	83.6 ± 15.3	88.9 ± 14.6	95.5 ± 18.4	91.3 ± 16.5	89.5 ± 21.8	77.3 ± 9.0	74.9 ± 17.3
	PLA	82.5 ± 19.9	93.9 ± 28.3	95.2 ± 25.9	101.7 ± 23.7	94.6 ± 31.2	95.4 ± 26.3	80.9 ± 19.6	73.9 ± 17.0
	COMP	73.3 ± 17.8	78.2 ± 13.4	92.8 ± 32.6	88.9 ± 15.1	91.8 ± 11.4	88.2 ± 9.4	73.7 ± 5.4	77.9 ± 15.9

		PRE	POST	60 min	120 min	180 min	240 min	24 h	48 h
CK (U/L)	CON	558 ± 172	572 ± 329	661 ± 234	669 ± 255	501 ± 162	603 ± 250	424 ± 118	460 ± 329
	PLA	794 ± 368	577 ± 288	786 ± 342	894 ± 439	752 ± 397	668 ± 367	612 ± 338	615 ± 439
	COMP	686 ± 401	557 ± 125	589 ± 200	709 ± 200	696 ± 180	835 ± 308	511 ± 242	689 ± 282

Time points are before exercise (PRE), immediately post-exercise (POST), 4 h recovery (60–240 min), and 24 and 48 h post-exercise. All data presented as mean ± SD.

^†^Significant time effect as compared with all other time points.

*Significant time effect as compared with PRE.

**significant time effect as compared with POST.

^#^Significant time effect as compared with 60 min.

^$^Significant time effect as compared with 24 h.

^§^Significant time effect as compared with 48 h.

^a^Small effect as compared with CON.

^b^Medium effect as compared with CON.

^c^Large effect as compared with CON.

^d^Small effect as compared with PLA.

^e^Medium effect as compared with PLA.

^f^Large effect as compared with PLA.

**Table 6:** Summary of all between-group effects for MS, TQR and thigh girth.

Measure	Group comparison	Change between	Mean difference in change		Standardised ES		Effect magnitude
			Absolute difference	± 95% CI	ES	± 95% CI	
MS (AU)	COMP vs. CON	PRE—120 min	0.71	1.50	0.68	1.30	Medium
		PRE—180 min	0.57	2.01	1.13	2.83	Large
		PRE—240 min	0.86	1.82	0.84	1.65	Large
		PRE—48 h	2.50	2.26	0.99	1.40	Large
	COMP vs. PLA	PRE—120 min	0.82	1.97	0.64	1.30	Medium
		PRE—180 min	0.96	1.99	0.70	1.32	Medium
		PRE—240 min	1.77	2.34	1.16	1.49	Large
		PRE—24 h	2.42	1.61	0.86	1.10	Large
		PRE—48 h	2.25	2.30	0.96	1.34	Large
TQR (AU)	COMP vs. CON	PRE—60 min	2.43	1.76	2.41	1.75	Large
		PRE—120 min	2.00	2.16	1.78	2.10	Large
		PRE—180 min	2.00	2.25	1.76	2.11	Large
		PRE—240 min	1.86	1.99	1.55	1.81	Large
		PRE—24 h	2.43	2.02	2.75	2.04	Large
		PRE—48 h	3.57	3.19	4.05	3.49	Large
	COMP vs. PLA	PRE—60 min	2.77	1.82	2.13	1.49	Large
		PRE—120 min	2.73	2.23	1.98	1.80	Large
		PRE—180 min	2.93	2.20	2.11	1.71	Large
		PRE—240 min	3.23	1.96	2.33	1.52	Large
		PRE—24 h	3.63	1.95	3.21	1.59	Large
		PRE—48 h	2.89	2.25	2.34	1.81	Large
Thigh girth (cm)	COMP vs. CON	PRE—60 min	1.57	0.32	0.29	0.05	Small
		PRE—120 min	1.47	0.24	0.27	0.04	Small
		PRE—180 min	1.40	0.24	0.25	0.04	Small
		PRE—240 min	1.33	0.21	0.24	0.03	Small
	COMP vs. PLA	PRE—60 min	1.68	0.33	0.35	0.06	Small
		PRE—120 min	1.47	0.27	0.31	0.05	Small
		PRE—180 min	1.35	0.28	0.28	0.05	Small
		PRE—240 min	1.36	0.25	0.29	0.05	Small

### Blood analysis

There were no interaction effects for plasma LDH (*F*_14, 152_ = 0.19, *P* = 0.999) or CK (*F*_*14, 152*_ = 0.43, *P* = 0.965), and no main effect of time for CK (*F*_7, 152_ = 1.30, *P* = 0.254). There were main effects of time (*F*_7, 152_ = 2.85, *P* = 0.009) for LDH. Specifically, LDH was increased at 60 min, 120 min, 180 min and 240 min as compared with baseline, 24 h, and 48 h (*P* < 0.05; Table 5).

### Thigh girth

There were no interaction effects (*F*_14, 152_ = 0.04, *P* = 1.000) or main effects of time (*F*_7, 152_ = 0.20, *P* = 0.985) for thigh girth circumference (Table 5). Effect size analysis revealed small effects for reduced thigh girth during the 4-h post-exercise recovery in the COMP group compared to the CON and PLA groups (Table 6).

## Discussion

The aim of this study was to assess the effects of sports compression tights on post-exercise blood flow, and to determine if the placebo effect was responsible for any acute performance or psychological benefits during the recovery from an eccentric lower-body resistance exercise session. Despite no significant group differences, the main findings from ES analysis were that compression tights increased blood flow during the 4-h post-exercise recovery period, and that this increase coincided with enhanced recovery of exercise performance and improved subjective ratings of soreness and recovery. Additionally, our findings suggest that compression was more effective in improving markers of venous return (ES range: 0.49 to 2.21), muscle blood flow (ES range: 0.44 to 1.15), exercise performance (ES range: 0.27 to 0.99), and subjective ratings of soreness and recovery (ES range: 0.64 to 4.05) as compared with both the placebo and control conditions. These results highlight that the ergogenic benefits associated with compression garments are paralleled with physiological alterations (e.g., increased blood flow), and that compression-induced improvements in exercise recovery are not explained by the placebo effect.

A novel component of this study was to monitor the effects of wearing sports compression tights for 4 h post-exercise on markers of venous return and muscle blood flow. The external pressure applied by sports compression is suggested to assist muscle pump action and enhance venous return by decreasing vein diameter, increasing venous flow velocity, and reducing venous pooling in the lower limbs^34–36^. Although no changes in venous diameter were present in the current study, there was medium to large effects for compression tights to enhance venous flow velocity during the 4-h recovery period. The increase in venous flow velocity is likely due to the shunting of blood from superficial veins to the deep venous system (i.e., popliteal and common femoral veins)^34–36^. Previous findings have observed compression-induced increases in similar markers of venous return at rest^37,38^. However, this is the first study to investigate the effect of compression tights on these markers of venous return post-exercise, and our findings suggest that these effects are maintained for up to 4 h post-exercise.

Our findings of enhanced markers of venous return coincided with large effects of attenuated EIMD symptoms including improved CMJ and IMTP performance recovery, TQR ratings and reduced muscle soreness and thigh girth swelling, which supports compression use for 4 h post-exercise as being beneficial for recovery. Furthermore, compression-induced increases in venous return may also serve as a protective mechanism against post-exercise hypotension, which can persist for several hours if individuals remain in a supine position during recovery after intense exercise^39^. Post-exercise hypotension, observed in trained^40^ and untrained^41^ individuals, is characterised by a reduction in blood pressure, and occurs due to a combination of an inactive muscle pump^42^, pooling of blood in previously active muscles^40^, decreased end-diastolic filling^43^, and reduced stroke volume^40^. The increase in venous velocity observed in this study, combined with compression garments resulting in a pronounced increase in stroke volume^33^, highlights the beneficial effects compression may have in preventing post-exercise hypotension. Furthermore, the enhanced venous return observed with compression may also serve to increase muscle blood flow via increases in arteriovenous pressure gradient and/or endothelial shear stress^37,44,45^.

Similar to markers of venous return, the effects of compression to enhance muscle blood flow were evident throughout the 4-h recovery period. Previous research has highlighted compression to enhance muscle blood flow during exercise^15,16^, and immediately post-exercise^18,19^. From ES analysis, the current study is the first to show that increased muscle blood flow is still present for 4 h post-exercise while wearing sports compression tights. Although the underlying mechanisms associated with compression-induced increases in muscle blood flow are less clear, and may be attributed to enhanced venous return^37,44,46^, it is frequently suggested that a myogenic response may provide an explanation^46,47^. The garment’s compressive effect is proposed to increase extravascular tissue pressure, subsequently reducing arteriolar transmural pressure and resulting in a reflex increase in arteriole vessel size (i.e., vasodilation)^46,48^. In turn, this leads to a decrease in arterial flow resistance, thus improving muscle blood flow^46,47^. Considering muscle blood flow is positively correlated with glucose uptake^49^ and rates of MPS^50^, compression may enhance the delivery of nutrients to the muscle, consequently enhancing the recovery and restoration process^10,15,31^. The only study to investigate the effect of compression garments on post-exercise nutrient delivery reported no effect of compression on glucose uptake^17^, likely due to the high level of pressure (37 mmHg) exerted to the limb (i.e., mechanically reduced muscle blood flow). Other post-exercise strategies promoting increases in limb (femoral artery) blood flow, such as hot water immersion^51^, have been reported to improve glucose metabolism^52^ and key markers of MPS and muscle hypertrophy^53^. However, these findings are not consistent^54,55^, and future research is required to investigate the effect of SCG on post-exercise nutrient uptake and rates of MPS due to their correlations with muscle blood flow.

A crucial component of this study, and the first in compression research, was the effective deception of participants administered the placebo intervention. To achieve this, participants in the PLA were given information sheets that highlighted similar benefits and mechanisms that are associated with compression (i.e., improved blood flow and reduced muscle damage/inflammation). After reading the information sheets, the PLA group had a higher belief rating for this intervention (3.9 ± 0.8) than the COMP group (3.0 ± 0.6). Thus, in support of our hypothesis, this study highlights that compression's performance and perceptual benefits are likely due to compression-induced physiological alterations and not a placebo effect. This is also supported by the increase in blood flow (i.e., venous flow velocity and muscle blood flow) coinciding with medium to large effects of improved performance and perceptual indices of recovery in the COMP group. These benefits were not present in the PLA or CON groups.

In the present study, the use of sport compression tights during recovery did not affect CK or LDH at any time point, in line with previous studies^32,56,57^. However, apart from a small increase in LDH concentrations during the 4-h recovery period, there were no significant increases in the measured blood parameters following exercise in all three groups. It is not uncommon for blood markers to remain unchanged following resistance exercise^58,59^, with the reliability of blood markers as an indicator of muscle damage questioned^60^. Several factors may explain the high variability in blood markers of EIMD and lack of effect reported in this study. A potential explanation for the lack of change in blood markers is that participants muscle were already in an exercise-induced damaged state, as evident in the high LDH and CK values pre-exercise. Although participants were asked to refrain from strenuous exercise 48 h prior to testing Session 2, an earlier exercise session (i.e., 72 h prior to testing Session 2) could be responsible for the elevated LDH and CK values pre-exercise. These blood markers can reach a peak level from 24 to 96 h following an exercise bout and may remain elevated for up to 7 days post-exercise^1^. Also, considering the magnitude of change in blood biomarkers of EIMD are typically greater in untrained than trained individuals^61^, as well as trained individuals possessing a more efficient mechanism of myofibrillar protein clearance following exercise^62^, the exercise intervention in the current study may not have been sufficient to elicit a significant response^58^ in this resistance-trained cohort. In addition, the inclusion of both male and female participants may have masked any impact of the exercise protocol on blood biomarkers, as females are reported to exhibit lower muscle damage marker activity following damaging exercise^63^. Despite the limited changes in blood markers, the exercise protocol resulted in elevations in muscle soreness and fatigue, and performance decrements.

The applied pressure from compression showed small effects in reducing thigh girth circumference during the 4-h recovery period, and potentially limiting the space available for fluid accumulation and swelling to occur^12^. Attenuating muscle swelling post-exercise may reduce the secondary inflammatory response and soreness^64^. The changes in muscle soreness and TQR reported in this study support this mechanism and are consistent with previous research^20,22,59^. In comparison, this is the first study to highlight that these benefits of compression garments on perceptual measures are not due to a placebo effect or prior belief in the efficacy of compression. The reduction in muscle soreness with COMP in this study, further highlights that compression tights used post-exercise may help limit muscle damage and decrease inflammation, thus improving exercise performance recovery.

Compression tights used for 4 h after an eccentric lower-body resistance exercise session appear to enhance (small to large effects) the recovery of CMJ and IMTP performance. Damage to the contractile elements of muscle following resistance exercise leads to oedema formation, resulting in muscle soreness and decrements in exercise performance^4,5^. The improved recovery of CMJ variables with COMP, observed in this study and consistent with previous research^59,64^, has been attributed to the enhanced repair of the muscle contractile elements^32^. In support of this, improved ratings of muscle soreness and exercise performance recovery were evident with COMP. Regarding IMTP, COMP was beneficial at 4 h post-exercise only (Force at 100 ms, Force at 200 ms, RFD 0 to 100 ms, RFD 0 to 200 ms). The application of compression is suggested to positively influence muscle fibre recruitment^65^ and muscle contraction efficiency^66^ due to reduced muscle movements^67^. Although speculative, the enhanced motor unit activation, important for maximising force development (i.e., RFD measures)^68^, may explain the enhanced RFD at 4 h post-exercise for COMP in the current study. However, this proposed mechanism requires further investigation.

Sports compression tights used for 4 h post a lower-body resistance exercise session appear to increase blood flow and improve perceptual and performance indices of exercise recovery. Furthermore, the addition of a successful placebo by deception in this study highlights that the benefits observed in the current study with compression were likely not due to a placebo effect. Therefore, sports compression tights might be a beneficial strategy to improve recovery when used for 4 h following an eccentric lower-body resistance exercise session. This finding might be valuable to athletes/individuals that may be in a supine position following exercise, training or competition for several hours (e.g., watching a movie, travelling via bus or plane) or when there is a short recovery period between training sessions (e.g., morning and evening sessions) or competitive events.

## Methods

### Participants

Thirteen males (mean ± SD: age, 24.9 ± 5.9 years; height, 179.5 ± 7.8 cm; body mass, 85.6 ± 10.7 kg) and nine females (mean ± SD: age, 27.3 ± 2.9 years; height, 167.4 ± 7.0 cm; body mass, 62.5 ± 11.8 kg) completed the study. The sample size was powered to detect a moderate difference (d = 0.54)^69^ in muscle blood flow with and without compression with an α value of 0.05 and 80% statistical power (G*Power Version 3.1.9.2; Universität Düsseldorf, Düsseldorf, Germany). Participants were required to be performing a minimum of two lower-body resistance exercise sessions a week, for a minimum of 6 months, to be eligible to participate. Written informed consent was obtained before participation. All participants were screened to ensure no contraindications were present for study participation, including cardiovascular risk factors (i.e., personal or family history of cardiovascular disease) and exercise capacity (e.g., musculoskeletal injury or joint pain). All procedures and methods were approved by the Victoria University Human Research Ethics Committee (HRE18-227) and performed in accordance with the relevant guidelines and regulations. The experimental approach was a between-subject, parallel-group design. Participants were assigned one of three recovery conditions in a randomised fashion and matched on belief in the interventions (as assessed during the familiarisation session) to control for the placebo effect. These conditions were sport compression tights [COMP, 4 males and 3 females (*n* = 7)], placebo by deception [PLA, 5 males and 3 females (*n* = 8)] or a passive control [CON, 4 males and 3 females (*n* = 7)]. A parallel-group design was chosen to avoid a repeated bout effect^70^ that would be present in a cross-over design. Participant characteristics are described in Table 7.

**Table 7:** Descriptive characteristics of participants in experimental groups (mean ± SD).

	COMP (*n* = 7)	PLA (*n* = 8)	CON (*n* = 7)
Age (years)	26.6 ± 5.2	26.9 ± 5.8	24.9 ± 5.1
Height (cm)	177.8 ± 3.5	172.6 ± 10.9	174.6 ± 11.5
Body mass (kg)	74.3 ± 14.2	76.2 ± 13.7	73.8 ± 19.3
BMI (kg/m^2^)	23.5 ± 3.8	25.5 ± 3.3	23.8 ± 4.0
Leg press 1 RM (kg)	260 ± 115	242 ± 78	244 ± 94
Quadriceps skinfold (mm)	8.8 ± 3.1	11.9 ± 5.8	11.2 ± 2.1

### Experimental overview

**Figure 4:**
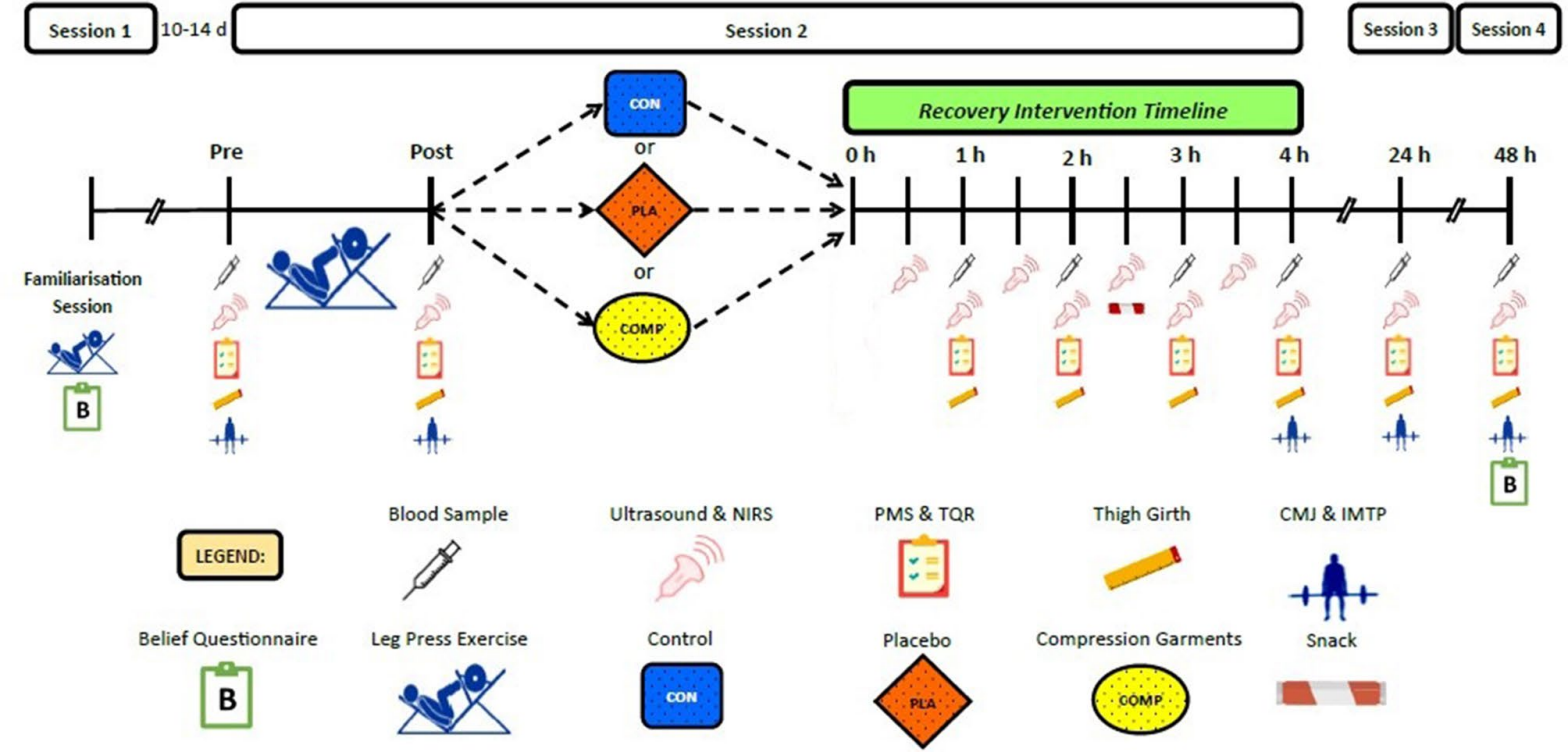
Schematic diagram of testing sessions.

Participants reported to the laboratory on four separate occasions (Fig. 4). Session one involved leg press one repetition max (1RM) testing, and familiarisation of the performance tests, blood flow measurements, and perceptual questionnaires. Anthropometric measurements of height, body mass, and quadriceps’ skinfold of the right leg were also taken. Following the reading of individual information sheets that highlighted the benefits of the COMP and PLA conditions for exercise recovery, participants completed belief questionnaires for both recovery interventions. Session one was conducted 10–14 days before session two.

Following a period of refraining from strenuous (< 48 h) or unaccustomed (< 7 days) exercise, participants reported to the laboratory in the morning for three experimental sessions. Participants were also asked to refrain from other exercise or recovery interventions (e.g., massage, water immersion) until the final testing session (session four) was complete. Session two started with pressure measurements of the sports compression tights (COMP only), followed by a 20-min supine rest period during which the sites for blood flow and thigh girth measurements were identified and prepared. Immediately after this, baseline measures of blood flow, perceptual questionnaires, thigh girth, and performance tests, as well as a venous blood sample, were collected. Participants then completed the leg press exercise protocol, and all baseline measures were repeated immediately after exercise. Following the post-exercise measures, participants performed their assigned recovery intervention (i.e., CON, PLA, or COMP) while supine for 4 h, and measurements were repeated at 30 (blood flow) or 60 (blood samples, perceptual questionnaires, thigh girth) min intervals. These measurements were collected in approximately 8 min at each time point. Performance tests were repeated at the end of 4 h supine rest only. Sessions three and four involved re-testing of baseline measures at 24 and 48 h post-exercise, respectively. At the end of the 48-h testing session, participants repeated the belief questionnaires in their completed recovery intervention. Testing sessions two, three, and four, started at the same time of day to minimise diurnal variations.

### Recovery information and belief questionnaires

During the familiarisation session, participants were given an information sheet on the efficacy of two recovery interventions, SCG and l-Arginine supplementation. These information sheets illustrated peer-reviewed data on the effectiveness of either SCG^71^ or l-Arginine^72^ in enhancing recovery post-exercise, with a particular focus on their capacity to improve blood flow and reduce the symptoms of EIMD. The benefits of l-Arginine^72^ were used to create a placebo by deception, with participants assigned to the PLA group falsely led to believe they were receiving L-Arginine tablets (detailed below) during 4 h of recovery post-exercise. A ‘belief’ questionnaire^27^ was used to assess the participants anticipated effectiveness of SCG and L-Arginine for exercise recovery. Participants were instructed to mark an ‘X’ on a 5-point likert scale, with 0 representing 'not effective at all' and 5 representing ‘extremely effective’. From this questionnaire, participants were assigned their recovery intervention (CON, PLA, or COMP). A participant was randomly assigned to either COMP or CON if they answered a higher belief in COMP than L-Arginine (PLA) after reading the information sheets, and vice versa for a higher belief in l-Arginine (PLA) (i.e., participant was randomly assigned to either PLA or CON). If a participant rated both interventions equally, they were then randomly assigned to one of the three recovery interventions. A similar ‘belief’ questionnaire was used at the end of testing to measure the participants’ perceived effectiveness of their completed recovery intervention (i.e., COMP or PLA).

### One repetition max testing

Prior to testing of 1RM, participants performed a standardised warm-up consisting of 5 min of cycling at 1 W per kg body mass, 10 repetitions of bodyweight squats, 10 repetitions on each leg of bodyweight walking lunges, high knee run over 20 m, heel kick run over 20 m, 3 submaximal counter-movement jumps (CMJ), and 1 maximal CMJ. Following the warm-up, participants performed two warm-up sets of the leg press protocol, with each set consisting of 10 reps with no weight and 5 reps at 50% of a participant’s self-estimated 1RM (Supplementary File). A participants 1RM was determined as previously described^73^. This 1RM was used to prescribe the workload for the lower-body resistance exercise session.

### Dietary control

Participants completed a 24-h diet diary before session two, and were asked to replicate this diet for the 24 h before sessions three and four. Participants were asked to refrain from caffeine and alcohol consumption (< 12 h) before all testing sessions. A snack (Aussie Bodies, Protein FX Super Bar, New Zealand) containing 25.6 g protein and 18.4 g carbohydrate was provided to each participant at 2 h and 30 min into recovery. The same snack was provided after session 3 to help maintain nutrition adherence post-testing.

### Venous return

Markers of venous return were measured at the popliteal and common femoral veins via Doppler ultrasound. The ultrasound examinations were performed using a CX50 Ultrasound System (Philips, USA), L12-3 MHz linear transducer and venous presets. Flow studies were performed by a single experience sonographer in a temperature-controlled (22 °C) environment. All measurements were obtained in a supine position and conducted as previously described^9^. Briefly, the common femoral veins were examined 2 cm above the saphenofemoral junction, with the compression garments turned down slightly to gain access. The popliteal veins were examined at the level of the knee crease. Prior to participants’ wearing compression tights, a small incision was made in the garment at the knee crease to create a window for the transducer to access the popliteal vein. Pilot data confirmed that the pressure of the compression tights was not altered by the small incision. The inner vessel transverse cross-sectional area (CSA; cm^2^), time-averaged mean blood flow velocity (V_mean_; cm/s) and time-averaged peak blood flow velocity (V_peak_; cm/s) measurements for popliteal and common femoral veins were obtained for at each time point (Fig. 4).

### Muscle blood flow

Muscle blood flow was assessed in the *vastus lateralis* muscle using near-infrared spectroscopy (NIRS) and multiple venous occlusion, as previously described^9,15^. Muscle blood flow was assessed immediately following markers of venous return measures at each time point (Fig. 4), with the average of the three occlusions (coefficient of variation (CV); 14.9 ± 10.9%) used for data analysis.

### Perceptual measures

Participants assessed their level of perceived muscle soreness (MS) via self-manual palpation of the gluteal and thigh muscles followed by rating their level of soreness using a visual analogue scale (0 = no soreness, 10 = extremely high soreness)^74^. Each participant’s level of perceived recovery and fatigue was assessed using the Total Quality of Recovery (TQR) scale, which is a scale from 6 (no recovery) to 20 (maximal recovery)^75^.

### Thigh girth

Girth measurements were taken at the midpoint of the right thigh to evaluate potential swelling of muscles resulting from exercise. In a standing position, the thigh midpoint was determined and marked as 4 cm distal to halfway between the greater trochanter and lateral epicondyle^27^. For the COMP group, the thigh midpoint was also determined with participants wearing the garments prior to 4 h supine rest, and the site marked with tape. Thigh circumference was measured around the thigh while the participant lay supine on the table, with the foot flat on the table surface and the knee at 90°^27^. Measurements were repeated twice at each time point (Fig. 4), with the average value used for analysis.

### Blood analyses

A 22-gauge indwelling venous catheter (Optiva IV Catheter 22G X 1′′, Smiths Medical, USA) was inserted into the antecubital vein. The catheter was kept patent with 0.9% saline (~ 3 mL; Pfizer, Australia) after each blood draw in session two. Blood samples at 24 and 48 h post-exercise were drawn via venepuncture (Winged Infusion Set 21G X 0.75′′, Smiths Medical, USA). Blood samples (~ 10 mL each) were collected into an EDTA tube (K2EDTA, Smiths Medical, USA) at each time point (Fig. 4). A portion (100 µL) of blood from baseline, 24 and 48 h blood samples were analysed immediately for total haemoglobin concentration (KX-21 N; Sysmex, Japan). The remaining whole blood was centrifuged at 1000×*g* and 4 °C for 10 min. The acquired plasma was stored at − 80 °C for subsequent analysis. All samples were analysed using commercially available kits for CK (Creatine Kinase Activity Assay Kit, Abcam, Melbourne, Australia) and LDH (Lactate Dehydrogenase Assay Kit, Abcam, Melbourne, Australia). All samples were analysed in duplicate. The intra-assay coefficients of variation were 2.6% for CK and 3.8% for LDH.

### Exercise performance testing

The CMJ and isometric mid-thigh pull (IMTP) performance tests were performed on a force platform (400S, Fitness Technology, Adelaide, Australia). Prior to all performance testing, participants performed the same standardised warm-up as completed before 1RM testing during session one. The CMJ test was chosen as it is a valid and reliable test for assessing the fatigue levels of lower body power^76^. Each participant completed five maximal jumps as previously described^76^, separated by 10 s of rest. The average of the five maximal jumps was used to derive jump height (m), relative peak force (N/kg), relative peak power (W/kg), and total duration (s).

The IMTP test is commonly used to assess fatigue and changes in maximum strength and rate of force development capabilities^77^. The IMTP protocol required participants to pull upward on an immovable bar for 3 s while standing on the force platform. The mid-thigh position and hip and knee angle positions were determined from previous protocols^78^. Two repetitions were performed, separated by a 2-min rest. A third repetition was performed if a > 250 N difference was observed between peak forces of the first two efforts. Force–time variables calculated from IMTP included absolute peak force (N), relative peak force (N/kg), force at 100 ms (N), force at 200 ms (N), rate of force development (RFD; ∆Force/∆Time) from 0 to 100 ms (N/s), and RFD from 0 to 200 ms (N/s). The average value reported across the two trials was used for analysis.

The raw force–time data for both CMJ and IMTP, sampled at 600 Hz, was collected using Ballistic Measurement System software (Fitness Technology, Adelaide, Australia). Raw force–time data was exported to and analysed in Microsoft Excel using spreadsheets specifically formulated for analysing CMJ^79^ and IMTP^80^.

### Exercise intervention

An eccentric focused leg press exercise protocol, consisting of 8 sets of 6 repetitions at 85% of 1RM, was used to induce lower-limb muscle damage. Participants assumed a seated position on the leg press machine (Hammer Strength Linear, Schiller Park, IL, USA) and placed feet shoulder-width apart and flat on the platform. Prior to the beginning of 8 sets, participants performed a total of two warm-up sets, 10 repetitions with no weight on the leg press machine and 8 repetitions at 50% of 1RM. Participants lowered the resistance platform slowly for a duration of 4 s (time recorded by the investigator), and then pushed the platform back to its starting position as quickly as possible by extending the legs for each repetition. A 3-min rest period was provided between each set.

### Recovery intervention

For the COMP group, lower-body sports compression tights (Refresh Recovery Tights, 2XU, Melbourne, Australia) were assigned to participants based on height and weight (manufacturer guidelines), with garments pressure measured at the beginning of session two via the Kikuhime device (mediGroup, Australia) at six different landmarks along the lower limb. The landmarks were 5 cm proximal to the distal border of the medial malleolus (A), 5 cm proximal to A (B), medial aspect of maximal calf girth (C), anterior aspect of the thigh 10 cm below landmark E (F), midpoint between the inguinal crease and the superior-posterior border of the patella (E) and 5 cm proximal to landmark E (F)^9^. The PLA group were given a sugar-free tablet (Hermesetas, Stevia Sweet 220 Tablets, Woolworths) from a de-identified container at 0, 1, 2, and 3 h following post-exercise performance testing. Participants assigned to CON did not participate in any recovery intervention but remained supine for 4 h.

### Statistical analysis

Data are presented as mean ± SD and were analysed using IBM SPSS Statistics (Version 19, IBM Corp., Chicago, IL, USA). Normality was confirmed using the Shapiro–Wilk test. Belief effect (COMP vs. PLA) was assessed using independent sample t-tests. Comparisons between recovery interventions were analysed using a two-way linear mixed model (ANOVA) with repeated measures for time, where the between-subject factor is the recovery intervention (CON vs. COMP vs. PLA), and the within-subject factor is time. Significance was set at *P* < 0.05. Where significant time or interaction (time x condition) effects were found, a Fisher LSD post-hoc analysis was used. Due to the small sample sizes, effect sizes (ES) analysis were incorporated as these are independent of sample size^81^, and were used to compliment null hypothesis statistical testing. The measures analysed included markers of venous return, muscle blood flow, perceptual measures (PMS, TQR), thigh girth, blood plasma measures (CK, LDH) and performance tests (CMJ, IMTP). Cohen’s conventions for ES (with 95% confidence intervals) were used for interpretation and were defined as small (0.20 to 0.49), medium (0.50 to 0.79), and large (≥ 0.80)^82^.

## Supplementary Information

Supplementary Figures.

## Data Availability

The datasets generated during and/or analysed during the current study are available from the corresponding author upon reasonable request.
