# Supplementary material for: Compression-induced improvements in post-exercise recovery are associated with enhanced blood flow, and are not due to the placebo effect
Source: Sci Rep. 2022 Oct 6;12:16762. doi: 10.1038/s41598-022-21029-2 (PMC9537593; doi:10.1038/s41598-022-21029-2)
Supplement: Supplementary file 1 — Supplementary Figures. [file 41598_2022_21029_MOESM1_ESM.pdf]

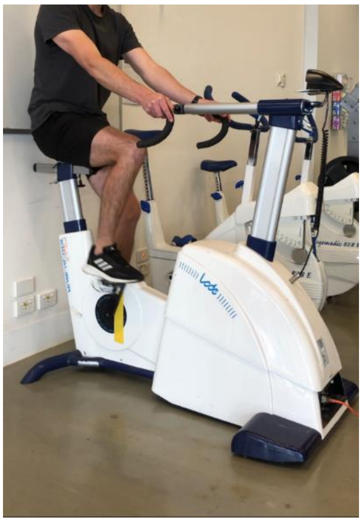

Supplementary Figures 1. Cycling at 1 W per kg body mass.

(i) (ii) (iii)

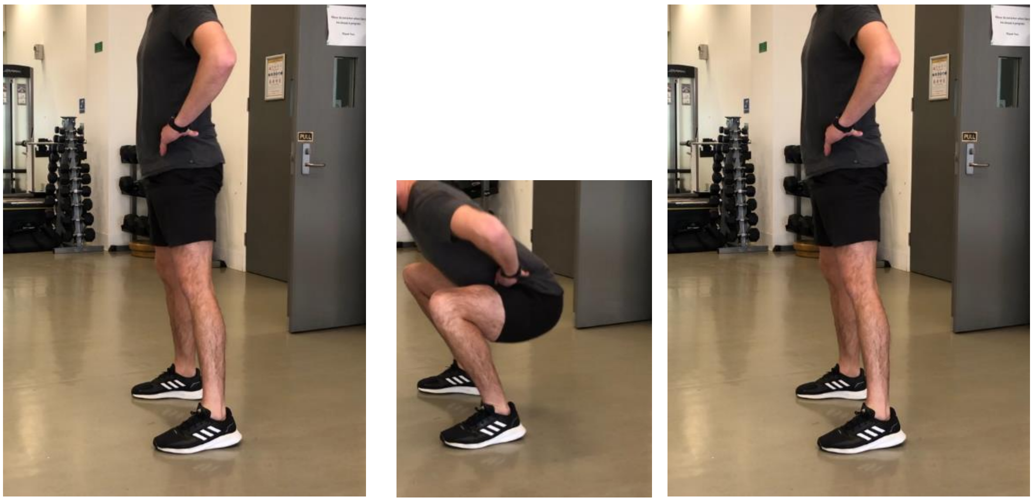

Supplementary Figures 2. Bodyweight Squats. (i) Starting position of a squat; (ii) Bottom position of a squat; (iii) End position of a squat.

(i) (ii)

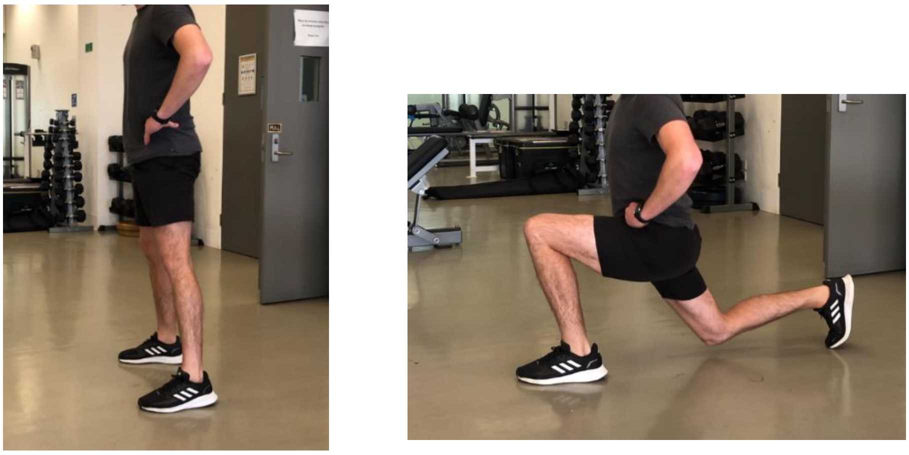

Supplementary Figures 3. Bodyweight Walking Lunges. (i) Starting position of a lunge; (ii) Bottom position of a lunge.

(i) (ii)

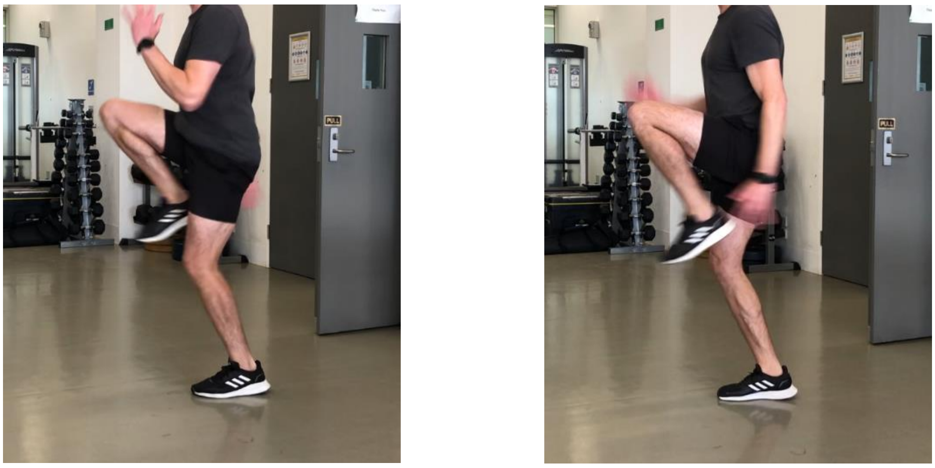

Supplementary Figures 4. Alternate High Knee Run. (i) Right high knee; (ii) Left high knee.

(i) (ii)

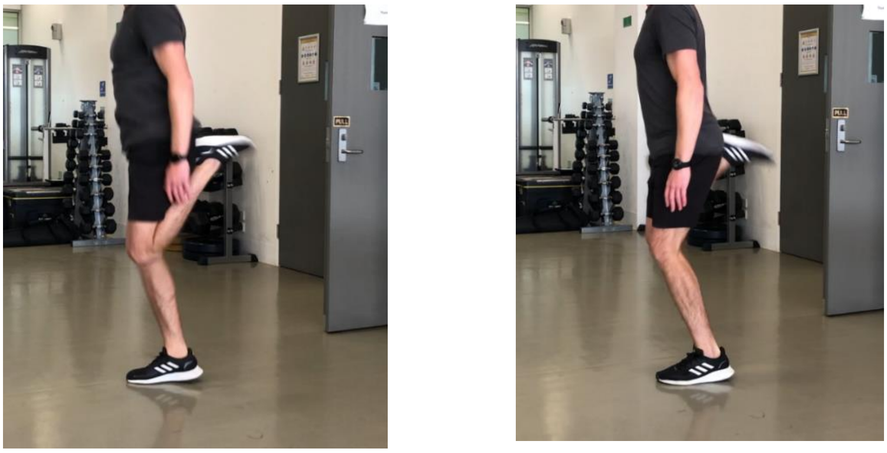

Supplementary Figures 5. Alternate Heel Kick Run. (i) Left heel kick; (ii) Right heel kick.

(i) (ii) (iii) (iv)

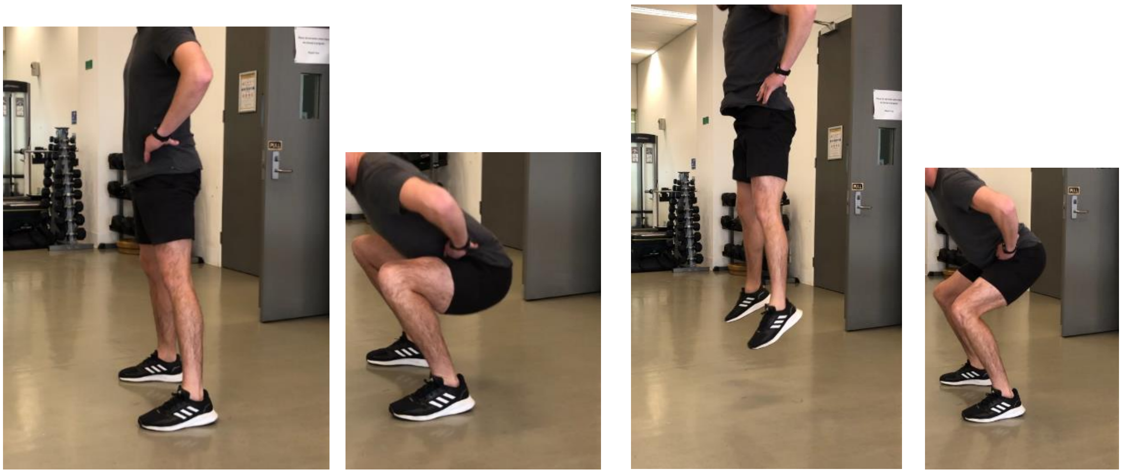

Supplementary Figures 6. Countermovement Jump (CMJ). (i) Start position of a CMJ; (ii) Bottom position of a CMJ; (iii) Top position of a CMJ; (iv) End position of a CMJ.

(i) (ii) (iii)

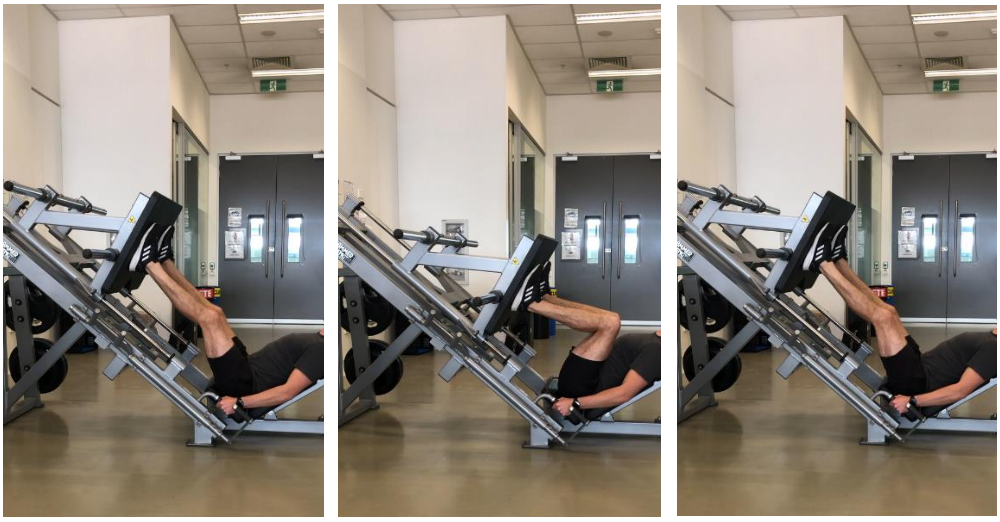

Supplementary Figures 7. Leg Press. (i) Starting position of a leg press; (ii) Bottom position of a leg press; (iii) End position of a leg press.
